# Milk-borne diseases through the lens of one health

**DOI:** 10.3389/fmicb.2023.1041051

**Published:** 2023-04-06

**Authors:** Sunandini Kapoor, Akhil Dhanesh Goel, Vidhi Jain

**Affiliations:** ^1^Department of Microbiology, All India Institute of Medical Sciences, Jodhpur, India; ^2^Department of Community Medicine and Family Medicine, All India Institute of Medical Sciences, Jodhpur, India

**Keywords:** one health, milk-borne disease, *Brucella*, pasteurization, cold chain

## Abstract

Reviewing “zoonotic diseases” classically brings to mind human infections contracted in close association with animals, where outdoor occupations and afforested lands usually play a key role in the epidemiological triad. However, there is a very common, yet overlooked route of infection where humans may not come in direct contact with animals or implicated environments. Milk-borne diseases are a unique set of infections affecting all age groups and occupational categories of humans, causing 4% of all the foodborne diseases in the world. The infection reservoir may lie with milch animals and associated enzootic cycles, and the infectious agent is freely secreted into the animal’s milk. Commercial pooling and processing of milk create unique environmental challenges, where lapses in quality control could introduce infective agents during downstream processing and distribution. The infectious agent is finally brought to the doorstep of both rural and urban households through such animal products. The domestic hygiene of the household finally determines human infections. One health approach can target preventive measures like immunization in animals, pasteurization and stringent quality control during the commercial processing of milk, and finally, hygienic practices at the level of the consumer, to reduce the burden of milk-borne diseases. This review hopes to draw the attention of policymakers to this unique route of infection, because it can be easily regulated with cost-effective interventions, to ensure the safety of this precious food product, permeating the life and livelihood of humans from all walks of life.

## Introduction

While milk processing has been a human tradition ever since humans domesticated animals, milk-borne diseases are rarely given their due importance as widely prevalent zoonotic diseases. Milk is an interesting source of infection because its consumption is widespread among all sections of society, and the resulting diseases can be prevented by minimal intervention. In this review, we aim at bringing to light the various ways by which milk aids in disease transmission and how we can prevent the transmission of these diseases by multisectoral coordination. We begin by discussing the burden of milk of borne diseases, followed by the interaction of environmental factors and pathogens in making this possible. Last but not the least, we attempt to outline possible approaches from veterinary, legal, administrative, and health perspectives to impede the transmission of milk borne diseases.

## Disease burden

Outbreaks related to infected milk consumption occur as sporadic cases in high-income countries while in low and middle-income countries, certain milk borne diseases are endemic, e.g., Brucellosis. Due to the uneven distribution of cases, and an array of pathogens that can be transmitted by milk, a definitive percentage summarizing the disease burden is lacking. However, to conjecture the same, the most credible evidence on diseases caused by milk and milk products is of the Global Burden of Foodborne Disease initiative by WHO which states that milk consumption causes 4% of all the foodborne zoonotic diseases in the world with low- and middle-income countries bearing the major brunt. This number represents only the tip of the iceberg. The actual disease burden may be masked due to the massive underreporting of cases throughout the world. Being a significant contributor to morbidity and mortality across the globe, dairy products have been estimated to be responsible for 20 DALY per 100,000 people in 2010. This accounts for approximately 12% of the food-borne disease burden associated with animal-source food (Li et al., 2019). Painter et al. (2009) estimated that dairy accounted for 9.7% of US Foodborne deaths each year. Unsurmountable economic losses to the measure of up to $ 77 billion in developed (Hoffmann and Anekwe, 2013) and countries and up to $95.2 billion in low and middle-income countries are incurred every year as a result of poor milk and farm hygiene (Jaffee et al., 2019). Table 1 summarizes the worldwide documented food-borne zoonotic disease outbreaks from the last decade. A thorough search for the outbreaks of various diseases due to milk consumption from 2013 to 2022 was done. Pathogen-focused and milk-related outbreaks were searched and information on the author, year, country, the number of cases, and the causative organism was determined. The keywords used to search for the outbreaks include milk borne outbreaks, Campylobacter in milk, STEC in milk, Brucellosis outbreaks, Non-Typhoidal Salmonella in milk, and *Listeria monocytogenes* in milk. Search engines used for the same include PubMed and Google scholar.

**TABLE 1:** Clinical features of milk borne pathogens in animals and humans.

Organism	Clinical presentation in animals	Clinical presentation in humans
*Campylobacter* spp.	Bloody diarrhea, vomiting, decreased appetite, fever	Blood and mucus in stool, fever, abdominal cramps
*Brucella* spp.	Abortion, still birth, vaginal discharge	Fever, weakness, malaise, hepatosplenomegaly, weight loss
Non-Typhoidal *Salmonella*	Diarrhea, dysentery, inflammation of joints, chronic pneumonia, asymptomatic carriage	Fever, abdominal cramps, vomiting, diarrhea
*Mycobacterium bovis* and *Mycobacterium avium* subsp. *paratuberculosis*	Weakness, loss of appetite, weight loss, intermittent hacking cough, diarrhea	Fever, night sweats, weight loss, chronic cough
*Coxiella burnetii*	Abortion, still birth, mastitis	Fever, chills, fatigue, headache, non-productive cough
*Helicobacter pylori*	Asymptomatic carriage	Gastritis, peptic ulcer disease, gastric carcinoma
Coxsackie virus	Blisters, abortion, fever, lameness	Blisters on hand, feet and mouth along with fever, sore throat
*Nocardia asteroides*	Pneumonia, mastitis, subcutaneous lesions, fever	Fever, weight loss, night sweats, chronic cough

Table 2 summarizes the various reports of outbreaks of milk-borne infections in the past decade.

**TABLE 2:** Outbreaks of milk borne zoonotic diseases from 2013 to 2022.

References	Year	Location	Pathogen	Number of people affected
Carpenter, 2013	2013	Alaska (Kenai Peninsula)	*Campylobacter*	4
Weltman et al., 2013	2013	Pennsylvania	*Campylobacter jejuni*	148
Davis et al., 2014	2014	Utah	*Campylobacter*	45
Johler et al., 2015	2014	Switzerland	*Staphylococcus aureus*	14
Hanson et al., 2019	2015–2016	Canada	*Listeria monocytogenes*	34
Kenyon et al., 2020	2016	North West England	*Campylobacter*	69
Mylius et al., 2018	2017	Germany	*E. coli* O103:H2	9
Robinson et al., 2020	2018	France	*Salmonella*	153
Boyd et al., 2021	2018	British Columbia	*E. coli* O121	7
Jenkins et al., 2022	2019	South Yorkshire, England	*E. coli* O157:H7	>20
Jones et al., 2019	2019	France	STEC 026	16
Gruber et al., 2021	2019	Pennsylvania, USA	*Yersinia enterocolitica*	109
Wang et al., 2020	2019	Guangdong, China	*Brucella melitensis*	30

## Understanding the epidemiological triad–Agent, host, and environment

**FIGURE 1:**
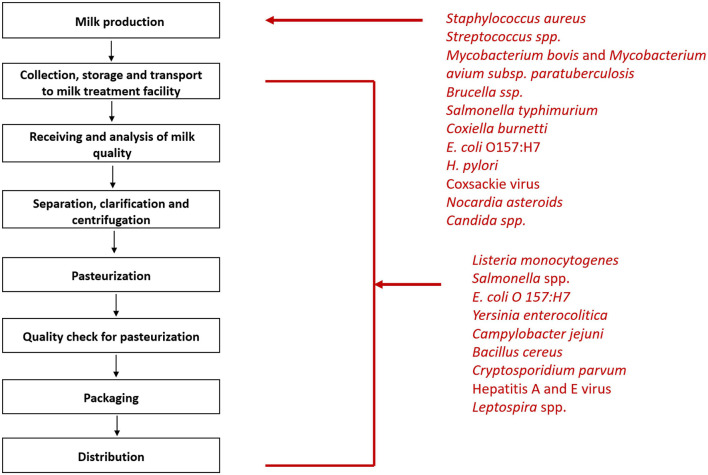
Steps of milk processing and list of pathogens that can enter milk at various stages of processing.

The microbiota of raw milk can be divided into three main categories- beneficial flora, spoilage flora, and pathogens. The beneficial flora of the milk includes *Lactococcus*, *Lactobacillus*, and *Leuconostoc* species. Spoilage organisms are the psychrotrophic organisms that flourish in milk even at low temperatures and cause degradation of milk components viz. *Pseudomonas, Acinetobacter, Serratia*, and *Aeromonas.* The third category is the pathogens which can make their way into the milk at any point- right from production to processing (Figure 1; Quigley et al., 2013). These include bacteria such as *Brucella melitensis* (Piao et al., 2020), *Campylobacter jejuni* (Davis et al., 2014), *Coxiella burnetii* (Signs et al., 2012), *Listeria monocytogenes* (Hanson et al., 2019), *Mycobacterium bovis* (Collins et al., 2022), Non-Typhoidal *Salmonella* (Robinson et al., 2020), and Shiga toxin producing *Escherichia coli* (Jones et al., 2019). The viral agents that may be transmitted by milk include Hepatitis A (Bidawid et al., 2000) and E virus (Huang et al., 2016), Norovirus (Yavarmanesh et al., 2015), Tick-borne encephalitis virus (Cisak et al., 2010). The uncommon bacterial and fungal agents include *Candida albicans*, *Candida krusei* (Lavoie et al., 2012), and *Nocardia* (Wahba et al., 2011) *asteroides*. Soil contamination of milk due to unhygienic handling practices can lead transmission of *Taenia solium via* milk. Other parasites making their way into the milk include *Ascaris lumbricoides* and *Trichuris trichiura* (Sujata et al., 2022).

Raw milk causes 840 times more illnesses and 45 times the hospitalizations caused by pasteurized milk (Costard et al., 2017). Taking developing countries into account, *Mycobacterium bovis*, one of the causal agents of human tuberculosis, is found in about 10–15% of the cases in developing countries (Ashford et al., 2001). It can remain viable in cheese and yogurt made from raw milk for up to 14 days and up to 100 days in butter making it highly transmissible (de la Rua-Domenech, 2006). World Health Organization estimates that *M. bovis* alone may have caused 143,000 new cases and 12,300 deaths in 2018 (Chakaya et al., 2022). Milk borne diseases would be incomplete without a mention of *Brucella* species. There is a lack of reliable data on the global burden of brucellosis. However, around 5,000,000 cases of Brucellosis are expected to occur each year which is very likely to be an understatement (Jamir et al., 2020). Another notorious pathogen is *Campylobacter spp.* which makes its way into the milk due to fecal contamination from infected organisms. Several cases of *Campylobacter* infection due to raw milk have been found in both developing and developed countries (Christidis et al., 2016).

Another notable fact is that a relatively younger set of the population is at a greater risk of acquiring infections due to raw milk consumption (Robinson et al., 2014). It can be attributed to a common myth that pasteurization alters the milk quality and decreases its nutritional value. This erroneous interpretation of pasteurization procedures by the masses has led to perilous consequences including hospitalization due to severe disease and even death (Costard et al., 2017). Contrary to the common belief, the availability and distribution of unpasteurized milk is higher in the recent years, particularly in developed countries, practically creating a ticking time bomb of milk borne infections (Kenyon et al., 2020).

Contrary to the belief that milk borne diseases are caused by the consumption of raw milk, pasteurized milk has also been implicated in several outbreaks. In the past decade, several reports of milk borne diseases have come into the picture. One notable report is of an outbreak caused due to *Yersinia enterocolitica* in contaminated pasteurized milk leading to 109 cases of enteritis in a youth summer camp in Pennsylvania (Gruber et al., 2021). A review of milk borne outbreaks in Canada from the year 2007 to 2020 showed 12 out of 32 outbreaks due to pasteurized milk resulting in 174 cases, 134 hospitalizations, and 17 deaths (Sebastianski et al., 2022). *Listeria monocytogenes* has been a common finding among outbreaks caused due to pasteurized milk. Several reports suggest that this organism has the capability of surviving pasteurization (Fleming et al., 1985). It has the potential to form biofilms on plastic and stainless steel surfaces, both of which are used to make milk storage equipment (Fleming et al., 1985). It has also been implicated in post-pasteurization contamination of milk *via* contaminated water and unsafe milk handling practices. Norovirus is another commonly found virus in pasteurized milk. Pasteurization is not a seal of guarantee ensuring the safety of milk for consumption. Improper pasteurization leading to the persistence of organisms and contamination of milk due to poor handling practices can inadvertently lead to contamination of milk. Maintaining the cold chain while transporting milk from the processing unit to the consumer is a relatively uncharted zone in terms of surveillance. Despite stringent measures to maintain the cold chain, milk storage temperatures are likely to increase irrespective of the season. Consequently, the standard plate count (SPC) of contaminating organisms will also rise with the increase in transit time (Franciosi et al., 2009).

Dry cow therapy is the treatment given to cows during the dry period, i.e., when the cow is not producing milk. There are two types of dry cow therapy given- Blanket dry cow therapy where systemic use of broad-spectrum antibiotics is done to prevent and treat infections and specific therapy where intra mammary antibiotic injections are given to treat infected mammary quarters (Kabera et al., 2021). Additionally, antibiotics are indiscriminately used as feed additives which ultimately make their way unaltered into the lacteal secretions. From their detection in milk for the first time in the 1960s, the levels of antibiotic residues have shown an upward trend over the years. β-lactam group of antibiotics has been found in milk in the highest number of studies followed by cephalosporins (Sachi et al., 2019). With the alarming rise in antimicrobial resistance and the havoc it has unleashed on humans, is it enough to have milk free from pathogens? Or should we move a step further and now view antibiotics in milk as a potential threat in itself?

## Human milk banking

Milk banking is the modern adaptation of the traditional practice of wet nursing. It is an effective way of providing human milk to infants who for various reasons do not have access to milk from their mothers. This is an excellent strategy as no other milk- whether from animals or artificially made can replace the benefits of human milk. However, milk banking faces several major challenges when it comes to worldwide implementation. Firstly, there is a paucity of global guidelines particularly for low- and middle-income countries. The dilemma regarding the classification of human milk as a nutritional source or a biological material of human origin causes issues in the legal aspects of milk banking. Secondly, each country has its own socioeconomic and cultural issues which may impact the collection, storage, and even consumption of human milk. Ethical issues, particularly the exchange of human milk for money are also necessary to be considered. Thirdly, evidence of the safety of various procedures such as pasteurization, storage, processing, etc., and their impact on milk quality is lacking and needs to be thoroughly researched (Corpeleijn et al., 2010). Human milk can serve as a conduit for several infections such as Q fever, Creutzfeldt- Jakob disease, etc. The origin of disease transmission *via* human milk banks begins with improper donor selection. As human milk is formed from blood, the donors are screened for all blood-borne pathogens. After screening and informed consent, the donor has to be counseled and instructed on the proper method of collection. After this, microbiological screening of milk should be done once before pasteurization and once after. Screening of organisms such as *S. aureus* and *E. coli* is done and the batch for which colony count exceeds 10^5^ CFU/ml is discarded. The milk is frozen for storage and is thawed in a water bath at 37°C (Infant and Young Child Feeding Chapter et al., 2014).

## One health approach for prevention of milk-borne infections

Milk-borne infections are a less-discussed subset of foodborne infections. However, this widespread route of infection is easily amenable to preventive and therapeutic measures. There are various research gaps, principally caused by the massive under-reporting of cases from low socio-economic agrarian economies. Further research is needed to devise easy and quick tests to detect the presence of pathogens in milk such as *Brucella* spp., *Coxiella burnetii*, *Salmonella* Typhimurium, *Campylobacter* spp., etc. Developing safe vaccines against agents such as *Coxiella burnetii*, *Mycobacterium bovis*, *Staphylococcus aureus*, etc., for milch animals can address emerging challenges in this field and the same can be seen as a lucrative research opportunity in the future.

**FIGURE 2:**
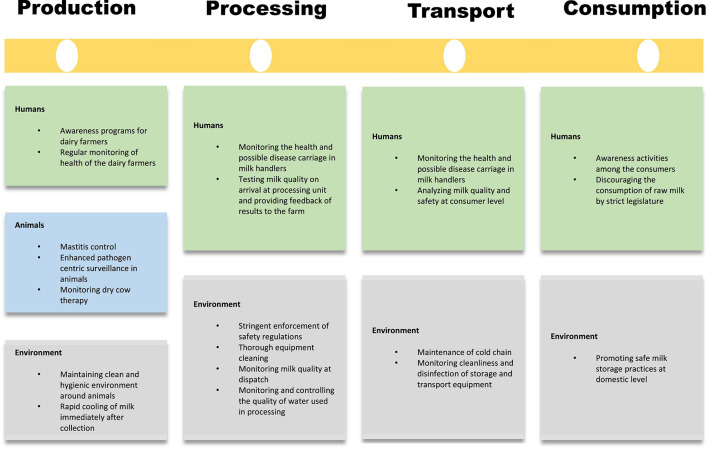
Four checkpoint approach to combat milk borne diseases at the level of production, processing, transport, and consumption.

Subsequently, a One Health approach to combating these diseases using a joint multisectoral effort is the need of the hour. Figure 2 summarizes various interventions that may be undertaken at the levels of milk production, processing, transport, and consumption. Comprehensive collaboration of veterinary, public health, and medical sciences can give the best results. The role of the strict legislature in controlling the sale of raw milk and ensuring adherence to safe milk processing and handling procedures should be ensured.

## Preventive strategies

### Health promotion

Awareness regarding milk borne infections among the masses is an arrow capable of striking multiple targets. Dairy farmers, milk handlers as well as consumers should be educated about common milk borne diseases, their potential hazards, and prevention strategies. Since outbreaks due to pasteurized milk are caused by pathogens that have human reservoirs, simple strategies like following hand hygiene practices and boiling the milk at home before consumption, are the most cost-effective strategies to prevent transmission of infection at the level of the consumer. Control of mastitis can be achieved by simple steps such as the use of proper milking machines, sanitization of teats pre and post-milking, vaccination against mastitis-causing organisms, lower density housing of animals, proper nutrition and maintenance of udder health during the dry period. Education must be given to dairy farmers to look for early signs and symptoms of diseases in the animals (Table 1) and the availability of the nearest veterinary services to get the animals treated.

### Specific protection

It is easy to understand that outbreaks due to raw milk are caused by pathogens that have animal reservoirs. Mandatory record keeping of vaccination status and health checkups of animals by dairy farmers is indispensable. Immunization of milch animals goes a long way in assuring the wellbeing of the animals. Firstly, it leads to the development of antibodies in the animal at levels enough to prevent the invasion of organisms in mammary glands. Secondly, it limits tissue damage and prevents poor yield of milk. Not only this, it should be ensured that follow-up vaccination is also provided to all animals (Nickerson and Ryman, 2023). Regular surveillance of fecal shedding of parasite eggs such as *Taenia*, coupled with routine de-worming can go a long way in improving milk yields and the overall health of the herd.

Another method of controlling the spread of these diseases is the slaughter of affected animals. Even though this approach has many legal and ethical issues attached to it, several countries have adopted the test “Test-and-slaughter” as a general policy against several diseases like Brucellosis and bovine TB. The animals are screened with the help of the Rose Bengal Plate Test and any positive results are then confirmed with Complement Fixation Test. Animals with confirmed positive results are then slaughtered or segregated from the rest of the herd. This approach has proved useful in keeping the prevalence of Brucellosis under control (Zamri-Saad and Kamarudin, 2016).

To prevent contamination of milk after it has been treated and ready for transport, monitoring of the cold chain (2–8°C) is an additional assurance in keeping organism counts to the minimum, even in the face of contamination from an external source.

### Early diagnosis

Early diagnosis of the disease is imperative in preventing further damage to the animal as well spread among the consumers. Easy to perform and quick diagnostic tests such as California Mastitis Test can help in the timely diagnosis and management of mastitis. Similarly, Somatic Cell Count (SCC) is also an important indicator of mastitis. The somatic cells present in milk are usually white blood cells. A cell count of more than 200,000 per ml of milk indicates infection. SCC of more than 400,000 per ml is considered unfit for human consumption (Deshapriya et al., 2019).

Conventional culture and antimicrobial susceptibility testing are needed for the detection of multidrug resistant bacterial contaminants of milk like Enterobacteriaceae (Yang et al., 2021). Molecular studies ranging from DNA extraction from milk specimens to qualitative and/or quantitative PCR-based pathogen detection (de Souza Ribeiro Mioni et al., 2019) are needed for the characterization of non-culturable pathogens like *Coxiella burnetii*. Serum specimens from cows may be analyzed for antibodies against suspected infective agents (Nogareda et al., 2012).

### Multisectoral collaboration

Most farmers and cattle owners are self-employed, small dairies may operate at the local village level and finally, commercial dairies receive pooled milk from various small-scale milk vendors. The industry further carries out complex downstream processing of milk. Transportation and household distribution are handled by a different chain of vendors. Therefore, coordination is needed at various levels, ranging from small farmers to large-scale industry owners and finally, end-user distribution channels. This coordination should aim toward a milk management system for the overarching goal of One Health.

### Role of legislation

Legislature regarding the sale of milk is variable among the countries of the world. Some nations have completely banned the sale of raw milk such as Canada. Several others continue to allow so either freely or in a controlled manner, i.e., allowing the sale of raw milk at farmers’ markets or *via* vending machines with a cautionary statement to boil the milk before consumption (ProCon.org, 2022). Proposing a uniformity of milk across the globe would be an impossible task due to several reasons:

1. In low- and middle-income countries, the sale of raw milk directly from dairy farmers to consumers is a major income source for many. Countries cannot ban the sale of milk through this channel as it promotes self-reliance among the dairy farmers.
2. The control over the sale of milk is not by a single body. E.g., in the USA. While the federal government has the authority to regulate the interstate sale of milk, the individual states retain control over the intrastate sale of dairy products but state law on the sale of raw milk is far from uniform.
3. Consumer demands for natural, organic, and whole foods, particularly those obtained from animals are on a rise. And with this, the clash between the government and the public has evolved.
4. No legislature can be implemented for an individual’s personal choice on milk consumption (David, 2012).

In an area where legislation may not prove to be useful, the role of the general public comes into play. Hence, the single and most effective tool to reduce the incidence of milk borne diseases is to encourage the involvement of local bodies in regulating the sale of raw milk.

## Conclusion

Milk-borne diseases are a unique set of infections affecting all age groups and occupational categories of humans. It is time that milk borne diseases are given their due importance as widely prevalent zoonotic diseases. The number of reported outbreaks of milk borne diseases seems to have reduced in the past decade or may simply be a consequence of underreporting of cases around the world. The need of the hour is enhanced surveillance strategies targeting milk borne diseases. This review hopes to draw the attention of local, national, and international policymakers toward this unique route of infection, because it is easily amenable to cost-effective interventions, ensuring the safety of this precious food product, and permeating the life and livelihood of humans from all walks of life.

## Author contributions

SK: preparation of all drafts of the manuscript. AG: development of idea and structure of the manuscript, figures, and tables. VJ: original idea, manuscript layout, and the final draft of the manuscript. All authors contributed to the article and approved the submitted version.
